# Current treatment status of fabry disease in South Korea: a longitudinal National health insurance service data-based study

**DOI:** 10.1186/s13023-025-03863-5

**Published:** 2025-07-10

**Authors:** DoHyeon Lee, Samel Park, Hyejin Yu, Eunjung Cho, Seung Seok Han, Eun Sil Koh, Byung Ha Chung, Kyung Hwan Jeong, Soo Jeong Choi, Eun Young Lee, Su Hyun Kim, Eun Hui Bae, Sunyong Yoo, Young Joo Kwon

**Affiliations:** 1 Department of Intelligent Electronic and Computer Engineering, Chonnam National University, Gwangju, South Korea; 2 Department of Internal Medicine, Soonchunhyang University Cheonan Hospital, Cheonan, South Korea; 3 Division of Nephrology, Department of Internal Medicine, Korea University College of Medicine, Seoul, South Korea; 4 Department of Internal Medicine, Seoul National University College of Medicine, Seoul, South Korea; 5 Department of Internal Medicine, Yeouido St. Mary’s Hospital, College of Medicine, The Catholic University of Korea, Seoul, South Korea; 6 Department of Internal Medicine, Seoul St. Mary’s Hospital, College of Medicine, The Catholic University of Korea, Seoul, South Korea; 7 Department of Internal Medicine, Kyung Hee University College of Medicine, Seoul, South Korea; 8 Department of Internal Medicine, Soonchunhyang University Bucheon Hospital, Bucheon, South Korea; 9 Department of Internal Medicine, Chung-Ang University Gwangmyeong Hospital, Chung-Ang University College of Medicine, Gwangmyeong, South Korea; 10 Department of Internal Medicine, Chonnam National University Medical School, Gwangju, South Korea

**Keywords:** Fabry disease, Enzyme-replacement therapy, Agalsidase-β, Agalsidase-α, Migalastat, Α-galactosidase A, Big-data

## Abstract

**Background:**

Fabry disease (FD) is an X-linked lysosomal storage disease caused by a mutation of the gene that encodes the α-galactosidase A enzyme. Treatment for FD is based on an enzyme replacement therapy (ERT), such as agalsidase-β, agalsidase-α, and migalastat. However, studies analyzing effects and outcomes of ERT in FD patients in South Korea are limited.

**Materials and methods:**

Treatment status and clinical outcomes of patients with FD in South Korea were investigated using data from the National Health Insurance Service (NHIS). The NHIS provides a comprehensive range of data across the entire Korean population, enabling an in-depth analysis of clinical outcomes associated with FD, including coronary composite heart disease, cerebrovascular disease, end-stage kidney disease (ESKD).

**Results:**

A total of 228 patients with FD were discovered. The diagnosis was earlier in males (*n* = 120) than in females (*n* = 108). Almost 90% of patients were treated only with intravenous agalsidase-β or -α. A total of 15 patients switched from agalsidase to migalastat. All clinical outcomes manifested at an earlier age in males than in females. Particularly, ESKD was more prevalent in males, both before and after diagnosis of FD. Patients who had ESKD at the time of FD diagnosis exhibited a higher hazard ratio (HR) for mortality (HR: 5.01, 95% confidence interval: 1.44–17.46).

**Conclusions:**

Our study showed the current treatment status and clinical outcomes in patients with FD in South Korea. Prior to the diagnosis of FD, a considerable number of patients had already reached ESKD, suggesting a lack of awareness of FD among clinicians. Given the higher mortality rate observed in patients with FD and accompanying ESKD, the necessity to improve awareness of FD is highlighted to facilitate early diagnosis.

**Supplementary Information:**

The online version contains supplementary material available at 10.1186/s13023-025-03863-5.

## Background

Fabry disease (FD) is an X-linked lysosomal storage disease caused by a mutation of GLA, a gene for α-galactosidase A (α-GLA) enzyme [1]. A deficit in the function of α-GLA results in a progressive disease alongside accumulation of glycolipids, such as globotriaosylceramide (GL-3) and globotriaosylsphingosine (lyso-GL-3), a derivative of GL-3 [2]. FD is a multi-faceted disease that causes damage in multiple organs, including heart, brain, and kidney [1, 3–5].

Since the introduction of enzyme replacement therapy (ERT) [6], two registries, namely the Fabry Outcome Survey (NCT03289065) and the Fabry Registry (NCT00196742), have been constructed to provide real-world evidence for ERT and increase our understanding of the natural history and progression of FD [7, 8]. Findings from these registries indicate that the ERT is initiated early in the disease course, the progression of disease can be significantly delayed, providing a fundamental basis for understanding the impact of ERT. Knowledge from these cohort studies have also resulted in several understandings, including effects of anti-drug antibodies and dose-dependent responses of treatment [9–11].

However, studies that comprehensively analyze effects and outcomes of ERT are notably rare in Korean literature. Most studies in South Korea have focused on clinical characteristics associated with Fabry disease for clues. Only one study has previously addressed long-term effects of ERT [12]. However, it only enrolled 19 patients. Recently, a report using National Health Insurance Service data has been published [13]. However, it only addressed major adverse cardiovascular events as a clinical outcome. In the light of the dearth of real-world evidence, the present study aimed to investigate treatment status in patients with FD, particularly drugs used and clinical outcomes experienced by FD patients in South Korea.

## Methods

The study protocol was reviewed and approved by the Institutional Review Board (IRB) of Soonchunhyang University Cheonan Hospital (Cheonan, Korea) (IRB No: SCH-2022-08-052). The requirement of informed consent was waived by the IRB due to a retrospective study design. The study was conducted in accordance with the principles of the Declaration of Helsinki. Raw data could be accessed via the National Health Insurance Service (NHIS) after obtaining approval.

### Study population

The objective of this study was to examine the current status of treatment for patients with FD in South Korea. To this end, data were curated from the NHIS, which collated medical big data in South Korea. The NHIS provides access to a comprehensive range of data, including claims data on details of prescribed medications and disease status as represented by the International Classification of Disease 10th revision (ICD-10) codes, as well as data from the National Health Examination programs, which encompassed the entire Korean population performed biennially [14]. The National Health Examination consists of health data, including laboratory and anthropometric measurements. Detailed description of NHIS data is available in our previous publication [15]. The estimated glomerular filtration rate (eGFR) was calculated using the Chronic Kidney Disease-Epidemiology Collaboration equation based on serum creatinine levels [16].

In the absence of a dedicated code in the ICD-10 system to specifically identify patients with FD, the code for other sphingolipidoses (E75.2) was employed to identify patients with FD. To distinguish between FD and other sphingolipidoses, an additional code (V117), an insurance code for a rare incurable disease, was utilized to indicate that patients had been enrolled in a copayment-decreasing policy. Once patients commence ERT, they are supported by this specific payment policy. Administration of ERT was screened to identify patients with FD. The date of diagnosis of FD was defined as the date of E75.2 occurrence in patients on ERT. The date of ERT initiation was defined as the date of the first administration of agalsidase-*β*, agalsidase-*α*, or migalastat.

### Clinical outcomes

Clinical outcomes were defined as follows: (1) ischemic heart disease, heart failure, and cerebrovascular disease (CVD) were defined by the occurrence of relevant ICD-10 codes on admission (I20– I25 for ischemic heart disease, I50 for heart failure, and I63 for CVD, respectively); (2) coronary angiography (CAG) with percutaneous coronary intervention (PCI) and pacemaker or implantable cardioverter-defibrillator (ICD) insertion were defined by the occurrence of relevant procedure codes; (3) end-stage kidney disease (ESKD) was defined by the occurrence of the ICD-10 code for renal failure (N18.5) with concomitant codes for copayment-decreasing policy on renal replacement therapy that lasted at least three months (V001 for hemodialysis, V003 for peritoneal dialysis, V005 for kidney transplantation); and (4) all-cause death was defined as death from any cause except for those associated with trauma or intoxication (S or T codes). The occurrence of these clinical outcomes was monitored from January 2002 to December 2021. Because serum creatinine levels were available in National Health Examination since 2009, annual eGFR data were collected from 2009 to 2021.

### Statistical analysis

Continuous variables are expressed as mean with standard deviation or as median with interquartile range (IR), as appropriate. Categorical variables are presented as a number and percentage. Continuous variables between groups were compared using either Student’s *t*-test or Mann-Whitney U test, as appropriate. Categorical variables were analyzed using either Pearson’s Chi-squared test or Fisher’s exact test. Non-parametric survival analysis was conducted using Kaplan-Meier curves with a log-rank test. Additionally, the Cox proportional hazards model with and without time-varying covariates was employed for survival analysis to calculate adjusted hazard ratio (HR) and 95% confidence interval (CI) related to all-cause mortality and other outcomes of interest. A linear model was utilized to calculate annual decline in eGFR (i.e., eGFR slope) for each patient.

## Results

### Study population

A total of 3,791 patients were identified with an ICD-10 code of E75.2. Among them, 1,307 patients exhibited an additional code of V117, indicative of a copayment decreasing policy. Drug codes for ERT, including agalsidase-*β*, agalsidase-*α*, and migalastat, were then searched. Ultimately, this study included 120 males and 108 females (Supplemental Figure S1).

Baseline characteristics of included patients are shown in Table 1. Male patients were diagnosed at a younger age than female patients, with an average age of 36.7 ± 15.2 years for males compared to 48.9 ± 16.0 years for females (*P* < 0.001). The median time interval between diagnosis of FD and initiation of ERT was 75 (IR: 43–151.5) days in males and 103.5 (IR: 53–218) days in females (*P* < 0.001). Approximately two-thirds of patients were diagnosed in the Department of Internal Medicine.

**Table 1:** Baseline characteristics of enrolled patients (*N* = 228)

Characteristics	Male (*N* = 120)	Female (*N* = 108)	*P* value
Age at diagnosed, years	36.7 (15.2)	48.9 (16.0)	< 0.001
Gap between diagnosis and initiation of ERT, days	75 (43–151.5)	103.5 (63–218)	< 0.001
Duration of observation, months	73.7 (40.3–151.4)	79.5 (32.1–107.1)	0.022
The initial department visited, n (%)			
Internal medicine	75 (62.5)	66 (61.1)	
Cardiology	30 (40.0)	37 (56.1)	
Nephrology	29 (38.7)	24 (36.3)	
Others	16 (21.3)	5 (7.6)	
Pediatrics	32 (26.7)	29 (26.9)	
Neurology	6 (5.0)	10 (9.3)	
Dermatology	5 (4.2)	0	
Others	2 (1.2)	3 (2.8)	
Treatment types			
aGAL β or α only			
No. of patients, n (%)	109 (90.8)	103 (95.4)	
No. of treatment	132 (36–241)	78 (32–181)	
aGAL β or α + miGAL			
aGAL → miGAL			
No. of patients, n (%)	10 (8.3)	5 (4.6)	
No. of treatment			
aGAL (no.)	103 (35–140)	122 (75–146)	
miGAL (days)	168 (126–322)	168 (126–434)	
aGAL → miGAL → aGAL			
No. of patients, n (%)	1 (0.8)		
No. of treatment			
aGAL (no.)^1^	340		
miGAL (days)^1^	126		

Continuous variables are represented as either mean (standard deviation) or median (interquartile range), as appropriate. Categorical variables are expressed as a number (percentage)

^1^As only one patient changed back from miGAL to aGAL, the total number of treatments and the number of days for which the treatment was prescribed were presented

Abbreviations: ERT, enzyme replacement therapy; aGAL, agalsidase; miGAL, migalastat

Nearly 90% of patients were treated exclusively with agalsidase-*β* or -*α*. Fifteen patients switched from agalsidase to migalastat. Of them, one patient switched back to agalsidase. The reason for this change back to agalsidase could not be determined. For the patient who switched back from migalastat to agalsidase, migalastat was prescribed for 126 days, equivalent to 256 days of treatment, as migalastat was taken every other day.

### Overall clinical outcomes

Table 2 shows an overall summary of clinical outcomes. The total number of patients who underwent CAG with PCI or pacemaker and/or ICD was insufficient for analysis, with only 6 cases involving CAG with PCI and 16 cases involving pacemaker and/or PCI. Therefore, ischemic heart disease, heart failure, CAG with PCI, pacemaker and/or ICD were combined to construct a new composite outcome, composite heart disease (CHD). CHD events were more frequently observed in female FD patients (103 cases in females vs. 93 cases in males, *P* < 0.001, Table 2 & Supplemental Table S1). Nonetheless, CHD occurred earlier in male FD patients both before the diagnosis of FD (47.6 ± 12.0 years in males vs. 58.7 ± 11.1 years in females, *P* < 0.001) and after the diagnosis of FD (35.7 ± 14.5 years in males vs. 48.1 ± 15.0 years in females, *P* < 0.001, Tables 2 and 3). For patients diagnosed with FD after CHD events (*N* = 107), the median time interval from FD diagnosis to the initiation of ERT was not significantly different between males and females (87 [IR: 57–146] days in males vs. 113 [IR: 64–215] days in females, *P* = 0.290, Table 3). The median interval between FD diagnosis and CHD occurrence was significantly different either between males and females (30.8 [IR: 7.2–66.0] months in males vs. 25.2 [IR: 2.5–54.4] months in females, *P* = 0.313). Both agalsidase and migalastat were used in five male FD patients and two female FD patients (Table 3).

**Table 2:** Overall summary of clinical outcomes

Characteristics	Male (*N* = 120)	Female (*N* = 108)
Composite heart disease^1^		
Before FD diagnosis, n (%)	43 (35.8)	46 (42.6)
Age at events, year (SD)^3^	47.6 (12.0)	58.7 (11.1)
After FD diagnosis, n (%)	50 (41.7)	57 (52.8)
Age at events, year (SD) ^3^	35.7 (14.5)	48.1 (15.0)
Months from FD diagnosis to event	30.8 (7.2–66.0)	25.2 (2.5–54.4)
Ischemic heart disease		
Before FD diagnosis, n (%)	37 (30.8)	38 (35.2)
After FD diagnosis, n (%)	23 (19.2)	30 (27.8)
Months from FD diagnosis to event	45.0 (10.4–125.9)	9.8 (1.5–71.5)
Heart failure		
Before FD diagnosis, n (%)	24 (20.0)	25 (23.1)
After FD diagnosis, n (%)	38 (31.7)	42 (38.9)
Months from FD diagnosis to event	42.9 (14.4–113.4)	36.0 (20.5–62.4)
CAG with PCI		
Before FD diagnosis, n (%)	3 (2.5)	2 (1.9)
After FD diagnosis, n (%)	0 (0)	1 (0.9)
Months from FD diagnosis to event	–	108.4
Pacemaker and/or ICD		
Before FD diagnosis, n (%)	3 (2.5)	2 (1.9)
After FD diagnosis, n (%)	8 (6.7)	3 (2.8)
Months from FD diagnosis to event	24.8 (2.1–50.5)	85.7 (56.0–108.1)
Cerebrovascular disease		
Before FD diagnosis, n (%)	12 (10.0)	16 (14.8)
Age at events, year (SD)	51.0 (9.5)	58.4 (13.5)
After FD diagnosis, n (%)	11 (9.2)	13 (12.0)
Age at events, year (SD)^1^	40.2 (8.1)	52.7 (6.6)
Months from FD diagnosis to event	42.2 (6.2–122.3)	68.0 (32.7–104.8)
End-stage kidney disease^1^		
Before FD diagnosis, n (%)	21 (17.5)	1 (0.9)
Age at events, year (SD)	48.2 (8.8)	40
After FD diagnosis, n (%)	12 (10.0)	1 (0.9)
Age at events, year (SD)	41.1 (9.5)	53
Months from FD diagnosis to event	42.2 (17.5–67.0)	87.86
All-cause death^2^		
After FD diagnosis, n (%)	15 (12.5)	3 (2.8)
Months from FD diagnosis to event	51 (18–79)	110 (14–118)
Total admission days, months	33.45 (15.11–71.06)	30.76 (3.1–64.4)

Overall clinical outcomes were summarized according to whether they occurred prior to or after the diagnosis of FD (referred to as “before FD diagnosis” and “after FD diagnosis,” respectively)

^1,2^A statistically significant difference was observed in the frequency of outcome among groups: those who did not experience the outcome, those who experienced the outcome prior to the diagnosis of FD, and those who experienced the outcome after the diagnosis of FD (by Fisher’s exact test). 1, *P* < 0.001; 2, *P* = 0.007

^3^A statistically significant difference was found in the age at diagnosis of FD between the two sexes. *P* < 0.001 by Student *t*-test

Abbreviations: FD, Fabry disease; CAG, coronary angiography; PCI, percutaneous coronary intervention; ICD, implantable cardioverter-defibrillator

**Table 3:** Comprehensive overview of composite heart disease

Characteristics	Before FD diagnosis				After FD diagnosis		
	Male (*N* = 43)	Female (*N* = 46)	*P* value		Male (*N* = 50)	Female (*N* = 57)	*P* value
Age at event, years (SD)	47.6 (12.0)	58.7 (11.1)	< 0. 001		35.7 (14.5)	48.1 (15.0)	< 0. 001
< 10 years, n (%)	–	–			1 (2.0)	–	
10–19 years, n (%)	1 (2.3)	–			6 (12.0)	3 (5.3)	
20–29 years, n (%)	2 (4.7)	1 (2.2)			9 (18.0)	6 (10.5)	
30–39 years, n (%)	6 (14.0)	–			13 (26.0)	4 (7.0)	
40–49 years, n (%)	16 (34.8)	10 (21.7)			12 (24.0)	14 (24.6)	
50–59 years, n (%)	12 (27.9)	14 (30.4)			6(12.0)	19 (33.3)	
60–69 years, n (%)	4 (9.3)	13 (28.3)			3 (6.0)	9 (15.8)	
70–79 years, n (%)	2 (4.7)	7 (15.2)			–	1 (1.8)	
≥ 80 years, n (%)	–	1 (2.2)			–	1 (1.8)	
Gap between diagnosis and initiation of ERT, days^1^					87 (57–146)	113 (64–215)	0.290
Gap between diagnosis and CHD, months^1^					30.8 (7.2–66.0)	25.2 (2.5–54.4)	0.313
Type of ERT							
Agalsidase-β *or -*α only					45	55	
Migalastat only					0	0	
Agalsidase-β *or* -α + Migalastat					5	2	
Total number of ERT							
Agalsidase-β *or* -α (no.)					130 (74–249)	157 (36–199)	
Migalastat (months)^1^					1, 9, 11, 11, 46	4, 62	

Continuous variables are presented as either the mean (standard deviation) or median (interquartile range), as appropriate. Categorical variables are expressed as number (percentage)

^1^Because only 5 males and 2 females were prescribed with migalastat, prescribed days of migalastat of each patient were described

Abbreviations: SD, standard deviation; ERT, enzyme replacement therapy; CHD, composite heart disease

CVD outcomes were observed in only 23 cases in males and 29 cases in females (Tables 2 and 4, Supplemental Table S1). Although the age of CVD onset was not different between sexes prior to FD diagnosis, CVD occurred at a younger age in male patients with FD than in female patients following FD diagnosis, with an average age of 40.2 ± 8.1 years in males and 52.7 ± 6.6 years in females (*P* < 0.001, Table 4).

**Table 4:** Comprehensive overview of cerebrovascular disease

Characteristics	Before FD diagnosis				After FD diagnosis		
	Male (*N* = 12)	Female (*N* = 16)	*P* value		Male (*N* = 11)	Female (*N* = 13)	*P* value
Age at event, years (SD)	51.0 (9.5)	58.4 (13.5)	0.103		**40.2 (8.1)**	**52.7 (6.6)**	**< 0.001**
20–29 years, n (%)	–	1 (6.3)			1 (8.3)	–	
30–39 years, n (%)	1 (8.3)	–			4 (41.7)	–	
40–49 years, n (%)	5 (41.7)	3 (18.8)			5 (25.0)	4 (30.8)	
50–59 years, n (%)	3 (25.0)	4 (25.0)			1 (16.7)	8 (61.5)	
60–69 years, n (%)	2 (16.7)	5 (31.3)			–	1 (7.7)	
70–79 years, n (%)	1 (8.3)	2 (12.5)			–	–	
≥ 80 years, n (%)	–	1 (6.3)			–	–	
Gap between diagnosis and initiation of ERT, days^1^					49 (41–125)	70 (60–266)	0.205
Gap between diagnosis and CVD, months^1^					42.3 (6.2–122.3)	68.0 (32.7–104.8)	0.270
Type of ERT							
Agalsidase β *or* α only					11	13	
Migalastat only					0	0	
Agalsidase β *or* α + Migalastat					0	0	
Total number of ERT							
Agalsidase β *or* α (no.)					168 (36–259)	198 (114–242)	
Migalastat (months)					0	0	

Continuous variables are presented as either the mean (standard deviation) or median (interquartile range), as appropriate. Categorical variables are expressed as number (percentage)

Abbreviations: SD, standard deviation; ERT, enzyme replacement therapy; CVD, cerebrovascular disease; aGAL, agalsidase; miGAL, migalastat

The median time interval between the diagnosis of FD and ERT was 49 [IR: 41–125] days in males and 70 [IR: 60–266] days in females (*P* = 0.205). Similarly, the median time interval between the diagnosis of FD and CVD events was 42.3 [IR: 6.2–122.3] months in males and 68.0 [IR: 32.7–104.8] months in females (*P* = 0.270). Neither interval showed a significant difference between males and females. All patients who had CVD events after the diagnosis of FD (*N* = 24) were treated with agalsidase only (Table 4).

As shown in Table 5, ESKD was more prevalent in male patients with FD (*N* = 33) than in female patients with FD (*N* = 2) (*P* < 0.001, Table 2 and Supplemental Table S1). Among the 35 patients who developed ESKD, 21 males and 1 female had been diagnosed with ESKD prior to FD diagnosis. The median time interval from FD diagnosis to the initiation of ERT was 60 [IR: 39.5–129] days in males and 64 days in females, while the interval from FD diagnosis to ESKD was 42.2 [IR: 17.5–67.0] months in males and 87.9 months in females, showing no significant difference between males and females (*P*_*Time_to_ERT*_ = 0.706 and *P*_*Time_to_ESKD*_ = 0.368). All patients who developed ESKD after a diagnosis of FD were treated with agalsidase only because migalastat was not indicated in patients with eGFR below 30 ml/min per 1.73 m^2^ (Table 5).

**Table 5:** Comprehensive overview of end-stage kidney disease

Characteristics	Before FD diagnosis				After FD diagnosis		
	Male (*N* = 21)	Female (*N* = 1)	*P* value		Male (*N* = 12)	Female (*N* = 1)	*P* value
Age at event, years (SD)	48.2 (8.8)	40	0.375		41.1 (9.5)	53	0.253
30–39 years, n (%)	4 (19.0)	–			6 (50)	–	
40–49 years, n (%)	9 (42.9)	1 (100)			3 (25)	–	
50–59 years, n (%)	5 (23.8)	–			3 (25)	1 (100)	
50–59 years, n (%)	3 (14.3)	–			–	–	
Gap between diagnosis and initiation of ERT, days^1^					60 (39.5–129)	64	0.706
Gap between diagnosis and ESKD, months^1^					42.2 (17.5–67.0)	87.9	0.368
Type of ERT							
Agalsidase β *or* α only					12	1	
Migalastat only					0	0	
Agalsidase β *or* α + Migalastat					0	0	
Total number of ERT							
Agalsidase β *or* α (no.)					160.5 (119.5–277.5)	183	
Migalastat (months)					0	0	

Continuous variables are presented as either the mean (standard deviation) or median (interquartile range), as appropriate. Categorical variables are expressed as number (percentage)

Abbreviations: SD, standard deviation; ERT, enzyme replacement therapy; ESKD, end-stage kidney disease; aGAL, agalsidase; miGAL, migalastat

**Fig. 1:**
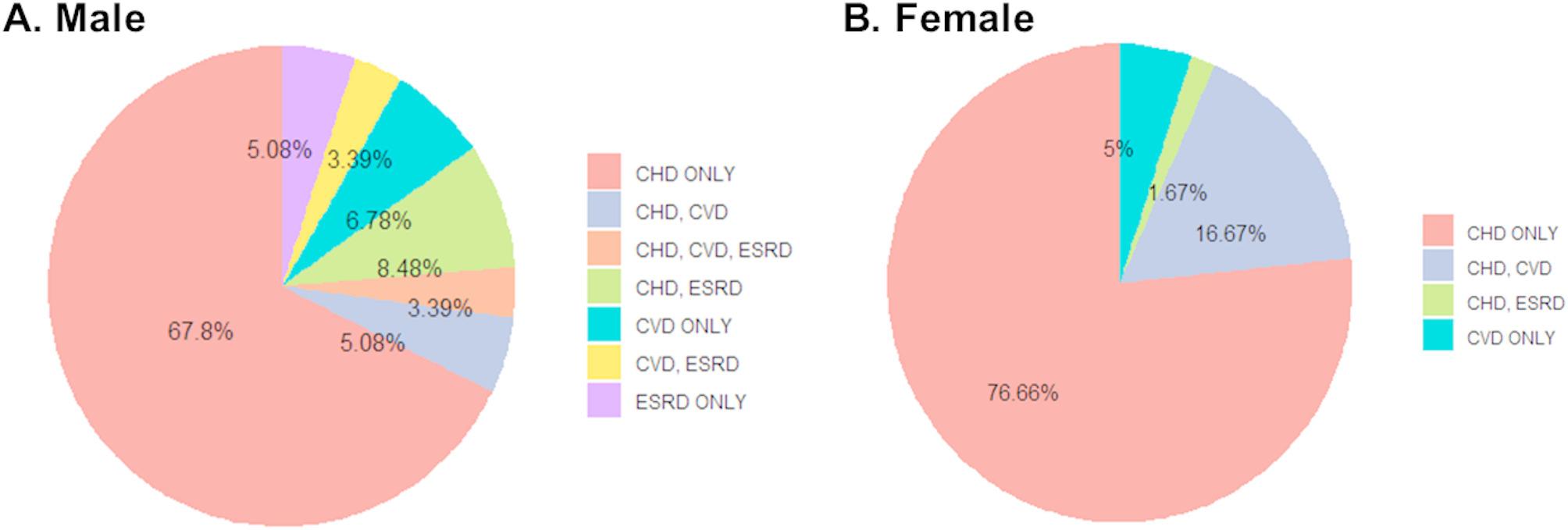
A pie chart to denote observed clinical outcomes The proportion of observed clinical outcomes is illustrated in a pie chart. A detailed overview of developed outcomes was provided for patients with two or more clinical outcomes Abbreviations: CHD, composite heart disease; CVD, cerebrovascular disease; ESKD, end-stage kidney disease

A single patient may present with a multiplicity of clinical manifestations. Consequently, the frequency with which each clinical manifestation occurred in each patient was determined. This could be a combination of two or more clinical manifestations. The most frequently observed clinical outcome was CHD in both males (67.8%) and females (76.66%) (Fig. 1). The second most frequent clinical outcome was CHD with ESKD in males (8.48%) and CHD with CVD (16.67%) in females. The mortality rate was significantly higher in male patients (12.5%) than in female patients (2.8%) (*P* = 0.007, Table 2).

### Estimations of kidney function declines using National health examinations

All Koreans are required to participate in the National Health Examination program biennially. This program encompasses a range of health assessment services, including anthropometric measurements, laboratory tests, and questionnaires. Each test is conducted in local clinics. Results of this National Health Examination program are then collected. Our data set included results of National Health Examinations. Among 228 patients enrolled in this study, 80 males and 94 females had at least one result from the National Health Examination program. Given the varying number of check-up results, initial and last results of the National Health Examination are presented in Table 6. Additionally, we sought to investigate whether there were any significant differences between males and females.

**Table 6:** Laboratory results from the National health examination in patients with Fabry disease who had two or more results

Variables	First results of National Health Examinations				Last results of National Health Examinations		
	Male (*N* = 80)	Female (*N* = 94)	*P* value		Male (*N* = 80)	Female (*N* = 94)	*P* value
Age group at diagnosis, years	41.2 (12.6)	51.2 (13.6)	< 0.001		41.2 (12.6)	51.2 (13.6)	< 0.001
Age at health screening, years	40.1 (9.5)	47.9 (11.9)	< 0.001		46.5 (10.8)	55.5 (13.1)	< 0.001
Months from diagnosis to NHE	-29.3 (-81.0–59.8)	-44.8 (-81.6– -1.6)	0.076		61.0 (0.6–135.8)	54.0 (13.6–92.8)	0.434
Months from ERT to NHE	-37.2 (-88.2–54.1)	-50.9 (-97.7– -14.8)	0.041		54.2 (-1.7–106.1)	44.1 (5.2–79.7)	0.402
Body mass index, kg/m^2^	22.5 (3.4)	22.9 (3.5)	0.437		23 (4.1)	23.2 (3.4)	0.633
Waist circumference, cm	78.9 (8.6)	75.4 (8.8)	0.011		81.4 (9.8)	77.1 (9.8)	0.005
SBP, mmHg	119.5 (16.0)	118 (14.2)	0.513		120.2 (13.6)	119.5 (14.7)	0.766
DBP, mmHg	74.1 (10.0)	73 (9.7)	0.448		73.8 (9.5)	72.4 (9.4)	0.341
Hemoglobin, g/dL	13.7 (1.4)	12.8 (1.1)	< 0.001		13.8 (1.5)	13.0 (1.2)	< 0.001
Fasting blood glucose, mg/dL	94.2 (20.1)	93.2 (13.4)	0.727		97.4 (21.4)	98.6 (34.9)	0.783
Total cholesterol, mg/dL	177.7 (44.7)	196.1 (40.9)	0.008		167.9 (45.8)	184.3 (38.6)	0.070
Triglycerides, mg/dL	117.1 (105.1)	106 (56.7)	0.418		87.6 (45.5)	105.9 (39.8)	0.046
cholesterol, mg/dL	67.3 (65.7)	59.4 (14.9)	0.317		61.8 (19.0)	58.1 (13.3)	0.282
LDL cholesterol, mg/dL	108.0 (110.4)	114.9 (39.2)	0.608		88.0 (33.4)	104.3 (33.8)	0.239
AST, IU/L	25.1 (11.0)	26 (9.95)	0.578		26.6 (11.9)	31 (13.6)	0.025
ALT, IU/L	21.3 (12.4)	20.2 (10.7)	0.542		22.8 (19.7)	23.8 (14.4)	0.725
GTP, IU/L	41.2 (42.2)	21.9 (16.7)	< 0.001		42.4 (42.9)	25.8 (22.8)	0.003
Creatinine, mg/dL	1.6 (2.1)	0.8 (0.9)	0.002		2.0 (2.4)	0.8 (0.4)	< 0.001
eGFR, mL/min per 1.73m^2^	84.5 (34.8)	92.1 (21.8)	0.095		75.5 (38.4)	88.3 (25.2)	0.012

Continuous variables are represented as either the mean (standard deviation) or median (interquartile range), as appropriate. Categorical variables are expressed as number (percentage)

Abbreviations: NHE, National Health Examination; ERT, enzyme-replacement therapy; NHE, National Health Examination; SBP, systolic blood pressure; DBP, diastolic blood pressure; LDL, low-density lipoprotein; AST, aspartate transaminase; ALT, alanine transaminase; GTP, gamma glutamyl-transferase; eGFR, estimated glomerular filtration rate

The precise year in which patients underwent National Health Examinations varied among individuals, making direct comparisons inappropriate. However, it was noteworthy that serum creatinine levels were higher in males at both the first screening (1.6 ± 2.1 mg/dL in males vs. 0.8 ± 0.9 mg/dL in females, *P* = 0.002) and the last screening (2.0 ± 2.4 mg/dL in males vs. 0.8 ± 0.4 mg/dL in females, *P* < 0.001) in National Health Examinations results.

Initial results indicated comparable eGFR values between males (84.5 ± 34.8 ml/min/1.73 m^2^) and females (92.1 ± 21.8 ml/min/1.73 m^2^) (*P* = 0.095). However, lower eGFR values were observed for male FD patients in last results than for females (75.5 ± 38.4 ml/min/1.73 m^2^ vs. 88.3 ± 25.2 ml/min/1.73 m^2^, *P* = 0.012) of the National Health Examinations, suggesting a rapid decline in eGFR in male FD patients. The observed decline in eGFR might be attributed to age-related variables employed in the CKD-EPI equation, rather than genuine changes in renal function.

**Fig. 2:**
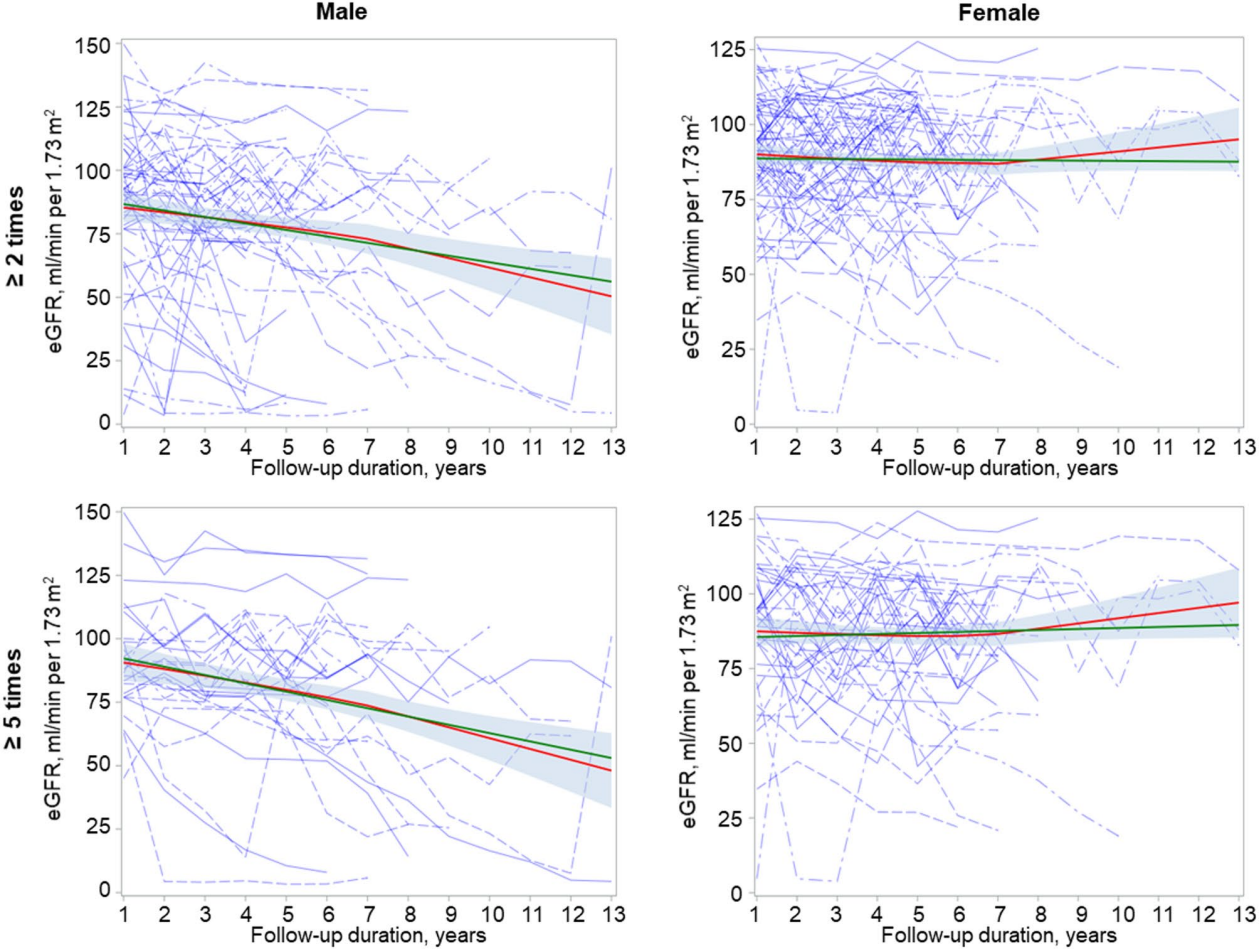
Estimated slopes of eGFR depicted by individual patient lines and fitted lines Blue dotted lines indicate the eGFR slope for each patient based on National Health Examination results. Red and green lines represent the fitted of eGFR line and its 95% confidence intervals (red lines, calculated using the loess method; green lines, calculated using the linear model)

To confirm this, we depicted eGFR declines and estimated slopes of eGFR using a linear model. Figure 2 presents estimated slopes of eGFR according to sex. As the number of patients who underwent National Health Examinations varied, patients with five or more results from National Health Examinations were separately subjected to sensitivity analysis. Estimated slope of eGFR was − 2.55 ml/min/1.73m^2^ per year (*P* < 0.001) for male FD patients and − 0.09 ml/min/1.73m^2^ per year (*P* = 0.835) for female FD patients. In a sensitivity analysis using patients with health screening ≥ 5 times, the estimated slope was − 3.27 ml/min/1.73m^2^ per year (*P* < 0.001) for males and − 0.34 ml/min/1.73m^2^ per year (*P* = 0.506) for females.

### Effect of gender on overall clinical outcomes

As FD is caused by a mutation of the GLA genes located on the X chromosome, the classic type of the disease, which is more severe, is more frequently observed in males. Genotype data were not available in this study. Therefore, we only investigated the extent to which male sex affected clinical outcomes using survival analysis with the Cox proportional hazard model.

When conducting research to investigate the occurrence of a specific disease, patients who have already been diagnosed with the disease prior to administration of a particular treatment are generally excluded. However, given that FD progresses with birth, the time interval between birth and the occurrence of a specific clinical outcome prior to the initiation of ERT should be considered when performing survival analysis. Furthermore, the initiation time of ERT and the duration of ERT varied among patients. To address these issues, analyses were conducted based on the following three models representing relevant hypotheses. Model 1 was a basic model that did not address whether ERT was administered or not because all patients in this study received ERT. In Model 1, age at initiation of ERT, sex, hypertension (HTN), and diabetes mellitus (DM) were adjusted for. History of HTN and DM was defined as a dichotomous variable indicating whether the patient had ever been diagnosed with either condition. In Models 2 and 3, covariates were adjusted to include effect of ERT after transforming ERT to a time-varying variable, in addition to variables used in Model 1. This was done to address variabilities in initiation time and duration. Distinction between Model 2 and Model 3 pertains to inclusion or exclusion of the time interval between birth and the occurrence of a specific clinical outcome prior to initiation of ERT in the model (Supplemental Figure S2).

Table 7 presents results of the analysis to examine the effect of sex on overall clinical outcomes, including CHD, CVD, ESKD, and all-cause death. Clinical outcomes including CVD, ESKD, and all-cause death, were comparable between models. Effects of sex on ESKD and all-cause mortality were statistically significant in all models. However, the effect of sex on CHD varied according to models. After adjusting for covariates, the effect of male sex on CHD was reversed in both Models 1 and Model 2 (crude HR: 1.56 [95% CI: 0.95–2.57] in both models; adjusted HR: 0.47 [95% CI: 0.26–0.84]) in Model 1 and 0.44 [95% CI: 0.25–0.77] in Model 2, Table 7).

**Table 7:** Effects of male sex on outcomes

Outcome	Model^1^	No. of Participants (M/F)	No. of Events (M/F)	Crude HR for male	Adjusted HR for male	*P*-value
CHD²	Model 1	75 / 61	34 / 38	1.56 (0.95–2.57)	0.47 (0.26–0.84)	0.011
	Model 2	75 / 61	34 / 38	1.56 (0.95–2.57)	0.44 (0.25–0.77)	0.004
	Model 3	120 / 108	79 / 85	1.95 (1.41–2.70)	1.06 (0.73–1.53)	0.778
CVD	Model 1	109 / 92	12 / 13	1.91 (0.85–4.30)	0.68 (0.25–1.86)	0.456
	Model 2	109 / 92	12 / 13	1.91 (0.85–4.30)	0.67 (0.25–1.81)	0.671
	Model 3	120 / 108	23 / 29	1.60 (0.91–2.82)	1.07 (0.54–2.09)	0.854
ESKD	Model 1	101 / 107	14 / 1	33.05 (4.30-254.18)	13.26 (1.60–110.02)	0.017
	Model 2	101 / 107	14 / 1	33.05 (4.30-254.18)	14.92 (1.80–123.94)	0.012
	Model 3	120 / 108	33 / 2	30.06 (7.15-126.41)	7.49 (1.62–34.73)	0.010
All-cause death	Model 1	120 / 108	15 / 3	14.39 (4.08–50.77)	8.65 (2.27–32.97)	0.002
	Model 2	120 / 108	15 / 3	14.39 (4.08–50.77)	9.50 (2.49–36.23)	0.001
	Model 3	120 / 108	15 / 3	14.39 (4.08–50.77)	9.50 (2.49–36.23)	0.001

^1^Model 1 was adjusted for the age at initiation of ERT, sex (male), HTN, and DM. Models 2 and 3 were adjusted for variables used in Model 1 and a time-varying variable, ERT. The difference between Model 2 and Model 3 was whether the time to outcome without ERT was added to the model or not. The history of HTN and DM was defined by whether the patient had ever been diagnosed with HTN or DM

^2^CHD was defined by the composite of ischemic heart disease, heart failure, pacemaker or implantable cardioverter defibrillator placement, or coronary angiography with percutaneous coronary intervention

Abbreviations: CHD,, composite heart disease; CVD, cerebrovascular disease; ESKD, end-stage kidney disease

### Risk factors for mortality in Fabry disease

**Fig. 3:**
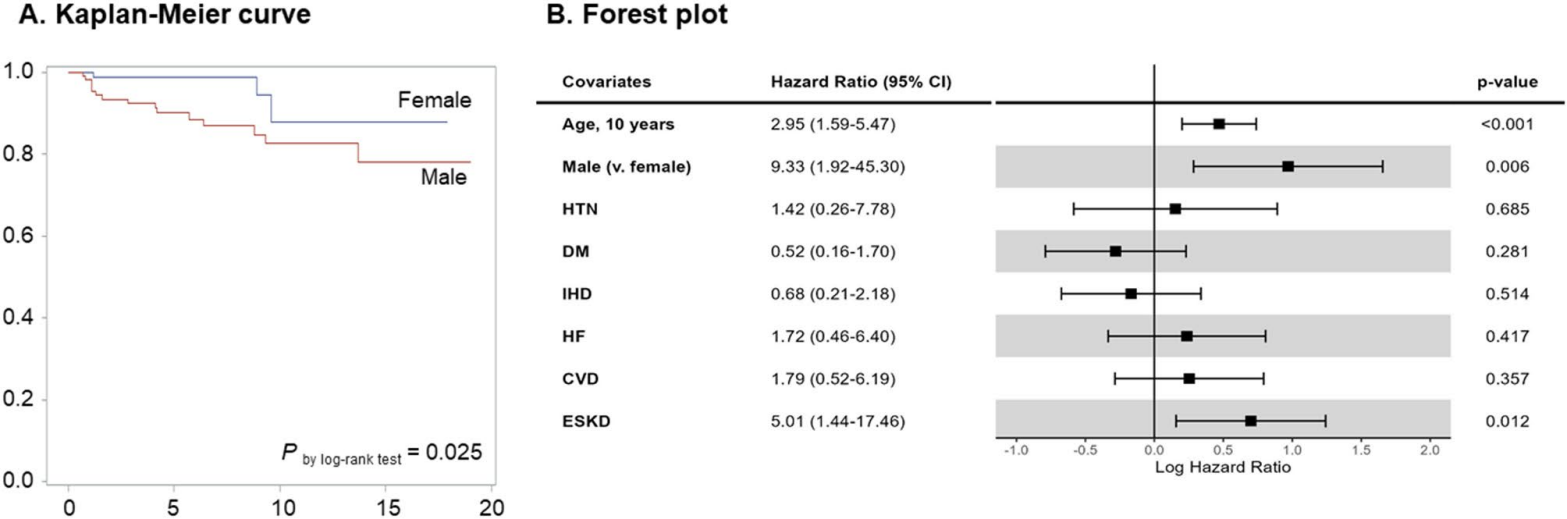
Risk factors for all-cause mortality A Cox proportional hazards model was used to investigate risk factors for all-cause mortality in Fabry patients on ERT. The model was adjusted for the age at initiation of ERT, sex (male), and comorbidities including HTN, DM, IHD, HF, CVD, and ESKD. All comorbidities were defined based on whether these comorbidities were diagnosed prior to ERT initiation Abbreviations: HTN, hypertension; DM, diabetes mellitus; IHD, ischemic heart disease; HF, heart failure; CVD, cerebrovascular disease; ESKD, end-stage kidney disease

Risk factors for mortality were also investigated. As illustrated in the preceding section, all-cause mortality was higher in male patients (Table 2). Non-parametric analysis of Kaplan-Meier curves and log-rank test revealed that all-cause death was more prevalent in males (*P* = 0.025, Fig. 3A). Results of survival analysis using the multivariable Cox proportional hazards model indicate that males, age, and ESKD were risk factors for all-cause mortality in patients with FD (Fig. 3B).

## Discussion

For the first time, the treatment status and clinical outcomes associated with ERT in patients FD in South Korea were determined, targeting almost the entire population of FD in the country. Descriptive statistics of treatment status are presented in as much detail as possible. As a result, representative clinical outcomes were closely examined and important risk factors for each outcome were analyzed. This study is the first to cover almost all FD patients in South Korea using the NHIS with National Health Examination Data. Findings of this study have significant implications for real-world clinical practice.

### Diagnosis of FD in South Korea

In this study, the average age at which FD diagnosis was made was earlier in males than in females (36.7 ± 15.2 years vs. 48.9 ± 16.0 years *P* < 0.001, Table 1). The age of FD diagnosis was comparable to that of the Fabry Registry (1,765 patients enrolled in December 2005, with 54% males) for males (mean age: 36.5 years), but earlier diagnosis was observed in females in the Fabry registry (mean age: 40.8 years) [17].

Such observed discrepancies in the age of diagnosis among females between our data set (the Korean data set) and the Fabry registry do not indicate a delay in the diagnosis of FD in females in South Korea. In accordance with Korean legislation, children are not permitted to be included when using NHIS data. Consequently, patients below the age of 20 at the time of data curation were excluded from this study (Supplemental Table S1). Among patients diagnosed with FD at a young age, only those who reached the age of 20 at the time of data curation were included in the analysis. This might be the reason for the discrepancy in the age of diagnosis of FD in females between the Korean data set and the Fabry registry. Furthermore, patients diagnosed recently in a pediatric department might have been underrepresented in our data set. However, in this study, two-thirds of the patients were diagnosed in the Deparment of Internal Medicine, comparable to the finding of a previous study [18].

### Treatment of patients with FD in South Korea

Characteristics of treatment status for FD in South Korea were inferred from the results of real-world clinical practices. Initially, nearly 90% of patients were treated with intravenous agalsidase-*β* or -*α* (Table 1), despite the burdensome nature of ERT due to its intravenous route and biweekly frequency. This is because migalastat is currently a secondary treatment option for FD in South Korea. Since migalastat is a secondary treatment option, none of the patients were eligible to commence treatment with migalastat (Table 1, Supplemental Table 3).

Furthermore, the time interval between the diagnosis of FD and the initiation of ERT was relatively brief in South Korea, with a duration of two months for males and three months for females (Table 1). This suggests that it is important to include FD as a differential diagnosis when practitioners encounter CHD, CVD, or CKD in young patients. As an earlier commencement of ERT has a positive impact on clinical outcomes [19], it is crucial for clinicians to be aware of the importance of suspecting FD and performing diagnostic tests.

### Involvement of heart and brain in patients with FD in South Korea

Primary clinical outcomes, namely CHD, CVD, and ESKD, are summarized in Tables 3 and 4, and 5, respectively. It was noteworthy that CHD was more prevalent in females than in males. Given the pathogenesis of FD, it was anticipated that complications would be more prevalent in males. Especially, males with classic FD show a higher rate of major cardiovascular events than non-classic FD and female FD patients [20]. Indeed, male patients exhibited a higher prevalence of CHD at an earlier age than in female patients both prior to and following FD diagnosis. Specifically, the mean age before FD diagnosis was 47.6 ± 12.0 in males and 58.7 ± 11.1 in females, while that after FD diagnosis was 35.7 ± 14.5 in males and 48.1 ± 15.0 in females (Table 3). Thus, we sought to elucidate underlying causes of the unexpected higher prevalence of CHD in female patients with FD such as genotype-phenotype differences in South Korea.

The crude HR for males on CHD was 1.56 [95% CI: 0.95–2.57] or 1.95 [95% CI: 1.41–2.70], based on aforementioned models (Table 7). The impact of ERT was not included in Model 1, as this model did not utilize administration of ERT as a covariate. In Model 2, the effect of ERT was too high to permit estimation of the HR (Supplemental Table S2). This might be attributed to the fact that all patients in Model 2 ultimately exhibited clinical outcomes. After adjusting for other covariates, being a female was identified as a risk factor for CHD in models 1 and 2, but not in model 3 (Table 7, Supplemental Table S2). However, ERT has a beneficial effect on patient outcomes. The results should indicate a decreased HR rather than an increased HR. The result of Model 3 was more likely to reflect real-world scenarios, as evidenced by the adjusted HR of ERT, which was 0.37 [95% CI: 0.24–0.57] (Supplemental Table S2). Additionally, results of model 3 indicated that CHD was prevalent in both sexes, showing no clear sexual predominance (HR: 1.06, 95% CI: 0.73–1.53, (*P* = 0.778). Interestingly, young age was identified as a risk factor, which might be attributed to the earlier onset of CHD in patients with the classic phenotype.

In 2021, 393 patients underwent ERT for FD (unpublished NHIS data), representing more patients than the number of patients observed in the present study (*N* = 228). The discrepancy between the two data could be attributed to the exclusion of patients with an age below 20 at the time of data curation who were not eligible for enrollment. These excluded patients might have exhibited a male gender and a classical phenotype given the early onset of FD. When these excluded patients could be considered together, results might be concordant with those previously observed. In South Korea, reimbursement for ERT became available for all ages in February 2014, whereas previously it was only available to those aged between 16 and 65 years (Supplemental Table S3). As a consequence of this insurance policy, the identification of patients with FD was slower in South Korea than in other countries, which might have influenced results of our study.

The observed incidence of CVD outcomes was less than anticipated. This could be due to the fact that the ICD-10 code for CVD outcomes was I63. Codes for cerebral small vessel disease, the most frequent form of neurologic manifestations in FD, were not included. The average age of CVD was significantly younger in male FD patients than in females after FD diagnosis (40.2 ± 8.1 years in males vs. 52.7 ± 6.6 years in females, *P* < 0.001, Table 4), although CVD occurred in both sexes without sexual predominance. Results of the survival analysis indicated that there was no statistically significant effect of sex on CVD outcomes (adjusted HR for males, 1.07 [95% CI: 0.54–2.09]), which was similar to findings for CHD (Table 7).

### Involvement of kidney in patients with FD in South Korea

Renal outcome was investigated utilizing of a specific code for ESKD and the eGFR slope derived from the National Health Examination data. It was noteworthy that a considerable number of patients were diagnosed with ESKD prior to being diagnosed with FD (21 males vs. 1 female, as detailed in Table 5). Although the age at which ESKD was diagnosed was slightly older than that observed in previous report [5], the younger age of dialysis initiation suggested that these patients progressed to ESKD due to FD. Because the genotype and the type of these patients (classic vs. late-onset) were not available, the cause of the difference in age of ESKD could not be determined. Moreover, the eGFR slope was found to be more rapid in males than in females, with a mean value of -2.55 ml/min/1.73m^2^ per year in males (*P* < 0.001) and − 0.09 ml/min/1.73m^2^ per year in females (*P* = 0.835), as illustrated in Fig. 2. The severity of proteinuria was not available, as the National Health Examination employed a urine dipstick analysis to measure proteinuria semi-quantitatively, which could not allow for a precise determination of proteinuria levels.

The interval between the diagnosis of FD and the commencement of ERT was relatively brief in South Korea (median 75 days in males and 103.5 days in females, Table 1). This indicates that there are few obstacles to the initiation of ERT in South Korea. Nevertheless, the high number of ESKD patients prior to diagnosis implied a lack of awareness of FD, particularly among nephrologists. A previous study demonstrated that, in South Korea, the mean duration from the onset of symptoms to FD diagnosis was approximately 10 years in adult males, compared to 1.5 years in pediatric males [21]. The involvement of kidney in FD could be neglected, as proteinuria and slightly elevated serum creatinine are often erroneously considered indicative of chronic glomerulonephritis rather than FD. Given that ERT is less effective in patients with advanced CKD (characterized by lower eGFR and severe proteinuria), early detection of FD is critical [22–24]. Furthermore, the observation of a higher mortality rate in patients who have already developed ESKD prior to FD diagnosis (Fig. 3) highlights that α-GLA activity and lyso-GL-3 levels should be considered in young patients with CKD with a family history. Among 897 patients receiving renal replacement therapy, 10 patients exhibited abnormal α-GLA enzyme activity or elevated lyso-GL-3, and one adult female was diagnosed with Fabry disease [25].

The present study has several limitations. First, the genotype was unknown due to the reliance on insurance claims data. The NHIS only had laboratory data that was performed in the general population for the diagnosis of early metabolic diseases and included body mass index, waist circumference, blood pressure, serum glucose, creatinine, and urine dipstick analysis. No clinical information was available on how the disease is diagnosed or how it progresses. For example, α-GLA levels, quantitative urine albumin measurements (albumin-to-creatinine ratio or 24-horr urine albuminuria), electrocardiogram, and echocardiography were not available. Therefore, the type of Fabry disease (classic vs. non-classic) could not be determined in our study. Although these limitations exist, it should be emphasized that in South Korea, all treatments for Fabry disease are strictly controlled by a national committee under the centralized NHIS, ensuring that our study accurately captured nearly all adult patients receiving ERT nationwide. Second, we were unable to adjust for the degree of proteinuria in this study. As mentioned earlier, only urine dipstick results were available, preventing the adjustment of our analysis based on the degree of proteinuria. Additionally, the legal age for enrollment of patients was below 20 years at the time of data collection, which further constrained the generalizability of our findings given that the FD is a genetic disease. Despite of these limitations, this study is notable for its comprehensive investigation of the treatment of FD and its outcomes in the Korean population with nationwide perspectives. Moreover, our results underscore the necessity for a multidisciplinary approach in managing patients with FD [26]. Fortunately, medical education programs on FD for primary care physicians and a neonatal screening program were initiated in South Korea in 2024. Future studies incorporating genotype and biochemical data including biochemical data are needed, particularly to assess untreated patients and clarify natural disease progression, especially in cases involving genetic variants of uncertain pathogenicity.

## Conclusions

We investigated ERT status and outcomes for FD patients in South Korea using data from the NHIS covering nearly all FD cases in the country. Nearly 90% of patients initially received intravenous agalsidase-β or -α, with migalastat available only as a secondary treatment. Our study found that males were diagnosed with FD earlier than females. We also identified a higher prevalence of cardiovascular and renal complications in males. The higher prevalence of ESKD before FD diagnosis underscored the importance of improvements in clinician awareness for early diagnosis of FD. Given the higher mortality rate observed in patients with FD and accompanying ESKD, the necessity to improve awareness of FD is highlighted to facilitate its early diagnosis.

## Electronic supplementary material

Below is the link to the electronic supplementary material.

Supplementary Material 1

Supplementary Material 2

## Data Availability

The data were acquired after appropriate request to National Health Insurance Service. The data could be accessed in permitted analysis centers for a specified period of time.
