# Supplementary material for: Current treatment status of fabry disease in South Korea: a longitudinal National health insurance service data-based study
Source: Orphanet J Rare Dis. 2025 Jul 10;20:355. doi: 10.1186/s13023-025-03863-5 (PMC12247461; doi:10.1186/s13023-025-03863-5)
Supplement: Supplementary file 1 — Supplementary Material 1 [file 13023_2025_3863_MOESM1_ESM.docx]

**Additional File 1: supplemental figures**

**
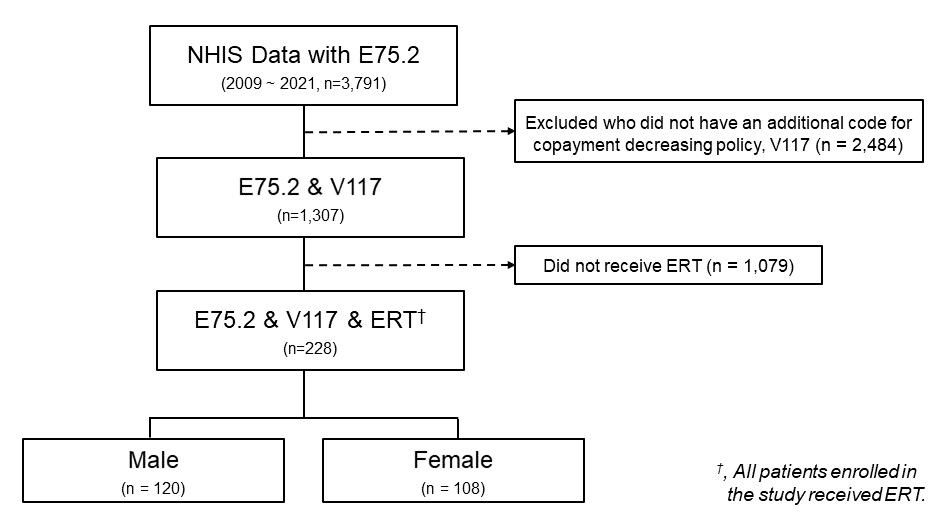
**

**Supplemental Figure S1. A depiction of the methodology for identifying and enrolling patients with Fabry disease.**

*Abbreviations:* NHIS, National Health Insurance Service; ERT, enzyme replacement therapy.


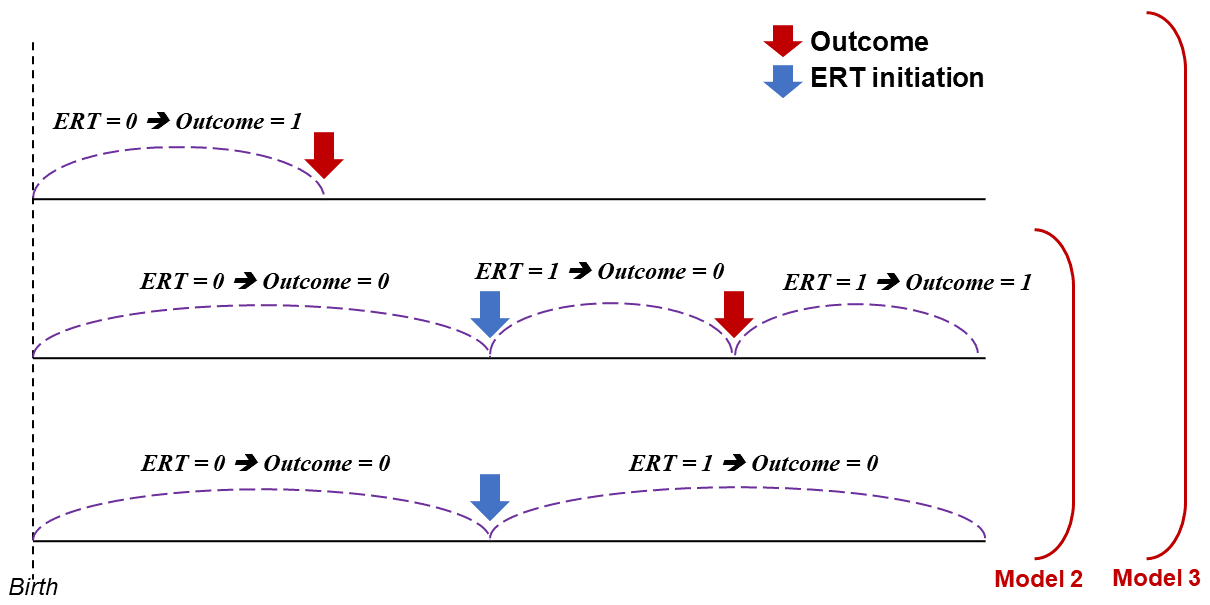


**Supplemental Figure S2. Simplified comparison between Model 2 and Model 3.**

Because all patients received ERT, Model 1 only adjusted for covariates including age at the initiation of ERT, sex, HTN, and DM. In Model 2 and Model 3, the duration of ERT was transformed to a time-varying variable, to be used in a time-varying Cox proportional hazard model. The difference between Model 2 and Model 3 was based on whether time interval from birth to the occurrence of any clinical outcome before initiation of ERT was included in the model.

*Abbreviations:* ERT, enzyme replacement therapy.
