# Supplementary material for: Current treatment status of fabry disease in South Korea: a longitudinal National health insurance service data-based study
Source: Orphanet J Rare Dis. 2025 Jul 10;20:355. doi: 10.1186/s13023-025-03863-5 (PMC12247461; doi:10.1186/s13023-025-03863-5)
Supplement: Supplementary file 2 — Supplementary Material 2 [file 13023_2025_3863_MOESM2_ESM.docx]

**Additional File 2: supplemental tables**

Supplemental Table S1. Proportion of patients diagnosed with Fabry disease, composite heart disease, cerebrovascular disease, and end-stage kidney disease according to their age by decade.

| **Characteristics** | **FD** | |  | **CHD** | |  | **CVD** | |  | **ESKD** | |
| --- | --- | --- | --- | --- | --- | --- | --- | --- | --- | --- | --- |
|  | **Male**  **(*N* = 120)** | **Female**  **(*N* = 108)** |  | **Male**  **(*N* = 93)** | **Female**  **(*N* = 103)** |  | **Male**  **(*N* = 23)** | **Female**  **(*N* = 29)** |  | **Male**  **(*N* = 33)** | **Female**  **(*N* = 2)** |
| Age at diagnosed, n (%) |  |  |  |  |  |  |  |  |  |  |  |
| < 10, years | 2 (1.7) | – |  | 1 (1.1) | – |  | – | – |  | – | – |
| 10 – 19, years | 17 (14.2) | 7 (6.5) |  | 7 (7.5) | 3 (2.9) |  | – | – |  | – | – |
| 20 – 29, years | 18 (15.0) | 9 (8.3) |  | 11 (11.8) | 7 (6.4) |  | 1 (4.3) | 1 (3.4) |  | – | – |
| 30 – 39, years | 29 (24.2) | 8 (7.4) |  | 19 (20.4) | 4 (3.9) |  | 5 (21.7) | – |  | 10 (30.3) | – |
| 40 – 49, years | 27 (22.5) | 28 (25.9) |  | 28 (30.1) | 24 (23.3) |  | 10 (43.5) | 7 (24.1) |  | 12 (36.4) | 1 (50.0) |
| 50 – 59, years | 20 (16.7) | 29 (26.9) |  | 18 (19.4) | 33 (32.0) |  | 4 (17.4) | 12 (41.4) |  | 8 (24.2) | 1 (50.0) |
| 60 – 69, years | 5 (4.2) | 18 (16.7) |  | 7 (7.5) | 22 (21.4) |  | 2 (8.7) | 6 (20.7) |  | 3 (9.1) | – |
| 70 – 79, years | 2 (1.7) | 8 (7.4) |  | 2 (2.2) | 8 (7.8) |  | 1 (4.3) | 2 (6.9) |  | – | – |
| ≥ 80, years | – | 1 (0.9) |  | – | 2 (1.9) |  | – | 1 (3.4) |  | – | – |

The number of patients in each age group is expressed as a number (percentage).

*Abbreviations:* FD, Fabry disease; CHD, composite heart disease; CVD, cerebrovascular disease; ESKD, end-stage kidney disease.

Supplemental Table S2. In-depth analysis for effect of sex on composite heart disease.

| **Outcome** | **Model** | **Events/**  **Participants** | **Variables** | **Crude HR** | ***P*-value** | **Adjusted HR^1^** | ***P*-value** |
| --- | --- | --- | --- | --- | --- | --- | --- |
| CHD² | Model 1 | 72 / 136 | Sex | 1.56 (0.95-2.57) | 0.079 | 0.47 (0.26-0.84) | 0.011 |
|  |  |  | Age, 10 yr | 0.26 (0.19-0.35) | <0.001 | 0.20 (0.14-0.29) | <0.001 |
|  |  |  | ERT | NA |  | NA |  |
|  |  |  | HTN | 0.85 (0.19-1.48) | 0.565 | 1.88 (0.96-3.70) | 0.067 |
|  |  |  | DM | 0.86 (0.54-1.39) | 0.537 | 1.24 (0.74-2.07) | 0.418 |
|  | Model 2 | 72 / 136 | Sex | 1.56 (0.95-2.57) | 0.079 | 0.44 (0.25-0.77) | 0.004 |
|  |  |  | Age, 10 yr | 0.26 (0.19-0.35) | <0.001 | 0.31 (0.22-0.45) | <0.001 |
|  |  |  | ERT | Too high^3^ |  | Too high^3^ |  |
|  |  |  | HTN | 0.85 (0.19-1.48) | 0.565 | 1.88 (0.96-3.70) | 0.040 |
|  |  |  | DM | 0.86 (0.54-1.39) | 0.537 | 1.25 (0.75-2.11) | 0.394 |
|  | Model 3 | 164 / 228 | Sex | 1.95 (1.41-2.70) | <0.001 | 1.06 (0.73-1.53) | 0.778 |
|  |  |  | Age, 10 yr | 0.43 (0.36-0.50) | <0.001 | 0.29 (0.23-0.37) | <0.001 |
|  |  |  | ERT | 1.73 (1.27-2.36) | 0.001 | 0.37 (0.24-0.57) | <0.001 |
|  |  |  | HTN | 0.83 (0.55-1.25) | 0.366 | 1.33 (0.84-2.10) | 0.232 |
|  |  |  | DM | 0.95 (0.70-1.30) | 0.757 | 1.12 (0.81-1.56) | 0.497 |

^1^ Model 1 was adjusted for the age at the initiation of ERT, sex (male), HTN, and DM. Models 2 and 3 were adjusted for variables used in Model 1 and a time-varying variable, ERT. The difference between Model 2 and Model 3 was whether the time to outcome without ERT was added to the model or not. The history of HTN and DM was defined by whether the patient had ever been diagnosed with HTN or DM.

^2^ CHD was defined by the composite of ischemic heart disease, heart failure, pacemaker or implantable cardioverter defibrillator placement, or coronary angiography with percutaneous coronary intervention.

^3^ In Model 2, patients who did not develop disease were censored from the survival analysis Therefore, all patients who received ERT had the disease, which resulted in the extremely high ERT variable.

*Abbreviations:* HR, hazard ratio; CHD, composite heart disease.

Supplemental Table S3. History of reimbursement for Fabry disease treatment in South Korea.

| **Drug name** | **Initial date of reimbursement coverage** |
| --- | --- |
| Agalsidase β | Sep 2002, in patients aged from 16 to 65 years with a decreased α-GLA activity.  Feb 2014, in patients having a decreased α-GLA activity or a confirmed pathogenic mutation |
| Agalsidase α | Aug 2014, in patients with a decreased α-GLA activity or with a confirmed pathogenic mutation |
| Migalastat | Mar 2019, in patients aged ≥16 years with amenable mutations after one year of ERT. |

*Abbreviations:* ERT, enzyme replacement therapy.
